# Characteristics of Cardiac Memory in Patients with Implanted Cardioverter-defibrillators: The Cardiac Memory with Implantable Cardioverter-defibrillator (CAMI) Study

**DOI:** 10.19102/icrm.2021.120204

**Published:** 2021-02-15

**Authors:** Kazi T. Haq, Jian Cao, Larisa G. Tereshchenko

**Affiliations:** ^1^Knight Cardiovascular Institute, Oregon Health and Science University, Portland, OR, USA; ^2^Medtronic, Inc., Minneapolis, MN, USA

**Keywords:** Cardiac memory, ICD, vectorcardiogram, ventricular pacing

## Abstract

This study sought to determine factors associated with cardiac memory (CM) in patients with implantable cardioverter-defibrillators (ICDs). Patients with structural heart disease [n = 20; mean age: 72.6 ± 11.6 years; 80% male; mean left ventricular ejection fraction (LVEF): 31.7 ± 7.6%; history of myocardial infarction in 75% and nonsustained ventricular tachycardia (NSVT) in 85%] and preserved atrioventricular conduction received dual-chamber ICDs for primary (80%) or secondary (20%) prevention. Standard 12-lead electrocardiograms were recorded in AAI and DDD modes before and after seven days of right ventricular (RV) pacing in DDD mode with a short atrioventricular delay. The direction (azimuth and elevation) and magnitude of spatial QRS, T, and spatial ventricular gradient vectors were measured before and after seven days of RV pacing. CM was quantified as the degree of alignment between QRS_DDD-7_ and T_AAI-7_ vectors (QRS_DDD-7_ –T_AAI-7_ angle). Circular statistics and mixed models with a random slope and intercept were adjusted for changes in cardiac activation, LVEF, known risk factors, and the use of medications known to affect CM occurring on days 1 through 7. The QRS_DDD-7_–T_AAI-7_ angle strongly correlated (circular r = −0.972; p < 0.0001) with a T_AAI-7_–T_DDD-7_ angle. In the mixed models, CM-T azimuth changes [+132° (95% confidence interval (CI): 80°–184°); p < 0.0001] were counteracted by the history of MI [−180° (95% CI: −320° to −40°); p = 0.011] and female sex [−162° (95% CI: −268° to −55°); p = 0.003]. A CM-T area increase [+15 (95% CI: 6–24) mV*ms; p < 0.0001] was amplified by NSVT history [+27 (95% CI: 4–46) mV*ms; p = 0.007]. These findings suggest that preexistent electrical remodeling affects CM in response to RV pacing, that CM exhibits saturation behavior, and that women reach CM saturation more easily than men.

## Introduction

Right ventricular (RV) apical pacing can cause pacing-induced cardiomyopathy,^1,2^ with nearly every fifth patient developing pacing-induced cardiomyopathy if the RV pacing burden is 20% or greater.^3,4^ Biventricular pacing and His-bundle pacing are more physiological but also more technically challenging pacing approaches. Any clinical decision made regarding the choice of pacing approach should be based on the evaluation of risks and benefits for each patient. Unfortunately, it remains largely unknown which factors (besides pacing burden) are associated with pacing-induced cardiomyopathy.

RV pacing changes the activation pathway and induces the complex repolarization phenomenon of cardiac memory (CM).^5^ CM is a form of cardiac electrical remodeling caused by altered myocardial stretch.^6^ Altered ventricular stretch and subsequent local cardiac angiotensin II release but not altered ventricular activation initiate CM.^7^ The T-wave changes affiliated with CM result from underlying changes in ion channels (eg, *I*_to_, *I*_kr_, and *I*_Ca,L_) and connexin 43 remodeling.^6^ CM can be fully reversible after a return to the normal activation pathway.^6,7^ All these facts suggest that the amount of CM that develops in response to RV pacing is likely associated with clinical outcomes. However, while molecular mechanisms of CM have been extensively studied,^5^ its translation into clinical practice has remained limited to date.^8^ The current clinical perception of CM is restricted by recognition of the T-wave inversion that develops after a period of altered ventricular activation once normal ventricular activation is restored, thus differentiating CM from ischemic T-wave inversion.^9^

Previously, CM was studied in patients undergoing pacemaker implantation.^10–13^ An interaction of RV pacing–induced CM with preexistent cardiac electrical remodeling [eg, after myocardial infarction (MI) and ventricular tachycardia (VT)] remains incompletely understood. The goal of this study was to determine factors associated with CM in patients receiving implantable cardioverter-defibrillators (ICDs). We hypothesized that preexistent cardiac electrical remodeling is associated with CM.

## Methods

The MATLAB (MathWorks, Inc., Natick, MA, USA) software code for electrocardiogram (ECG) data analysis and a subset of deidentified data is provided at https://physionet.org/physiotools/globalelectricalheterogeneity/, https://github.com/Tereshchenkolab/Origin, and https://github.com/Tereshchenkolab/cardiacmemory. Fully deidentified data (digital ECG files) are openly provided at https://github.com/Tereshchenkolab/cardiacmemory.

### Study population

The present Cardiac Memory with ICD (CAMI) prospective study was sponsored by Medtronic (Minneapolis, MN, USA). Study participants were enrolled between November 6, 2007 and April 7, 2010, at Beth Israel Deaconess Medical Center (BIDMC) and the data analysis for this investigation was performed at Oregon Health & Science University (OHSU). This study was approved by the institutional review boards of BIDMC and OHSU. All study participants signed informed consent forms before entering the study.

Eligible patients for inclusion included adults older than 18 years of age who had received a Medtronic market-released dual-chamber ICD with chronically implanted (for at least three months, ie, ≥ 90 days) Medtronic RV leads with a superior vena cava (SVC) coil and an RV ring electrode (Sprint Fidelis, Sprint Quattro Secure 6947, Sprint Quattro 6944, etc.; Medtronic) for approved indications. It was required that the RV tip be implanted in the RV apex and that patients were in sinus rhythm with 1:1 AV conduction at baseline.

Exclusion criteria were (1) a history of unstable angina pectoris within the last three months unless treated by coronary intervention; (2) an inability to tolerate DDD pacing or AAI pacing due to subjective discomfort, heart failure (HF), or another reason; (3) antitachycardia pacing or shock therapy from the ICD for spontaneous tachyarrhythmia episodes in the last three months; (4) more than 1% of RV pacing in the last three months (confirmed by the ICD device interrogation); (5) New York Heart Association class III or IV congestive HF; (6) left ventricular (LV) ejection fraction (LVEF) of less than 20%; (7) baseline ECG abnormalities (eg, complete left bundle branch block, T-wave inversion secondary to LV hypertrophy) precluding the expression of CM; and (8) an inability to return to the study center for follow-up assessments.

### ECG recording and pacing protocol: induction of cardiac memory

At the baseline study visit, resting supine 12-lead ECGs were recorded using a MAC 5000 electrocardiograph (GE Marquette, Milwaukee, WI, USA) in AAI and DDD modes with a short AV delay at a rate 10% faster than the presenting sinus rhythm. Then, ICD devices were programmed in the DDD mode with a short (100–120 ms) paced and sensed AV delay and a lower rate as clinically indicated.

The second study visit was conducted after seven days of ventricular pacing (VP) and the percentage of VP data was collected. During the follow-up visit, resting 12-lead ECGs were recorded first in DDD mode, then in AAI mode. The pacing rate during ECG recording during a follow-up visit was the same as that during the baseline ECG recording.

### Measurement of cardiac memory on body-surface vectorcardiograms

The raw digital 12-lead ECG signal (sampling rate: 500 Hz, amplitude resolution: 1 μV) was analyzed. The Kors matrix was used to transform the 12-lead ECG into an orthogonal XYZ ECG. All 10-second ECG recordings were reviewed and all beats were manually labeled. Ectopic beats, fusion beats, and artifact-distorted beats were excluded from the analysis.

Signal processing of the digital ECG signal was composed of the following steps: first, we used the Kors transformation matrix to transform the 12-lead ECG signal into the orthogonal XYZ ECG.^14^ To construct a single-lead median beat, we aligned all beats included in the analysis by the maximum absolute of the first derivative (maximum |dV/dt|), as previously described.^15^ Next, we constructed a time-coherent global XYZ median beat. Only the X-lead was used to perform the alignment, while corresponding time points on the Y- and Z-leads were taken to derive the time-coherent global XYZ median beat.^15^ We used a previously developed algorithm^15^ to identify the electrically quiet origin point of the heart vector as the flattest isoelectric line on the vector magnitude signal. The accuracy of the automated algorithm for the origin point detection was verified by two investigators. In two of 80 ECGs (2.5%) the location of the origin point was manually corrected. Both investigators were in full agreement.

Following detection of the heart vector origin point,^15^ four types of median beats were constructed as follows: AAI-mode atrial-paced ventricular-sensed (VS) (APVS) beats recorded on study day 1 (AAI-1) and day 7 (AAI-7), respectively, and DDD-mode atrial-paced VP (APVP) beats recorded on study day 1 (DDD-1) and day 7 (DDD-7), respectively.

Fiducial points (QRS onset and offset and T-wave offset) were automatically detected on the vector magnitude signal. Accuracy of the fiducial point detection was verified using a visual aid. In three of 80 ECGs (3.8%), fiducial points were manually corrected. There was complete (100%) agreement between the two investigators.

Spatial peak and area QRS, T, and spatial ventricular gradient (SVG) vectors were defined as previously described^16^ and their direction (azimuth and elevation) and magnitude were measured. The scalar value of SVG was measured by the sum of the absolute QRST integral (SAI QRST)^17,18^ and the QT integral on the vector magnitude signal (iVMQT).^16^

**Figure 1:**
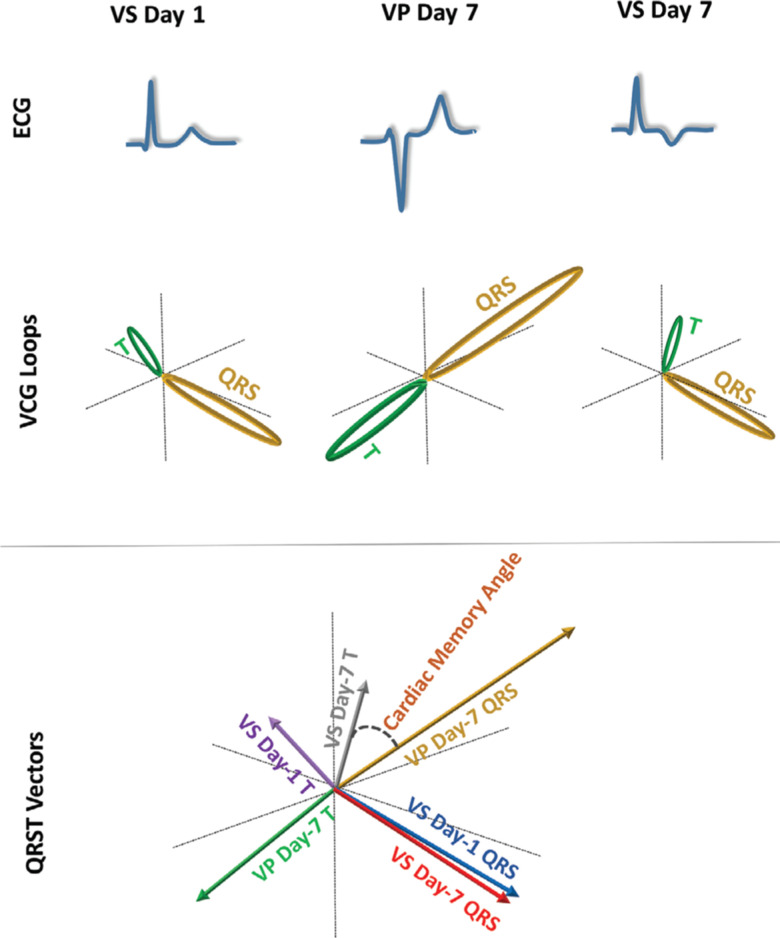
Schematic presentation of VCG loops and vectors. ECG: electrocardiogram; VCG: vectorcardiogram; VS: ventricular-sensed; VP: ventricular-paced.

CM was quantified after seven days of VP as the degree of alignment between the VP QRS vector (QRS_DDD-7_) and the VS T vector (T_AAI-7_), measured as the QRS_DDD-7_–T_AAI-7_ angle **(Figure 1)**. Changes in ventricular repolarization were assessed as the T_AAI-1_–T_AAI-7_, T_DDD-1_–T_DDD-7_, T_AAI-1_–T_DDD-1_, or T_AAI-7_–T_DDD-7_ angles.

The difference in ventricular activation between the VS and VP QRS vectors was measured by the QRS_AAI-7_–QRS_DDD-7_ angle. To eliminate an error due to possible variations in the placement of ECG leads between days 1 and 7, CM angles were measured on same-day recordings. To assess the possible error due to the placement of ECG leads on two different days, we measured the spatial angles QRS_DDD-1_–QRS_DDD-7_ and QRS_AAI-1_–QRS_AAI-7_ and determined an agreement between angles QRS_AAI-7_–QRS_DDD-7_ and QRS_AAI-1_–QRS_DDD-1_.

### Statistical analyses

The distribution of all variables was evaluated. A quantile–quantile plot was used for checking normality. Normally distributed continuous variables were presented as mean ± standard deviation (SD) values. A paired t-test was used to compare normally distributed vectorcardiogram (VCG) parameters in different pacing modes at baseline and after seven days of VP.

Circular statistics were used to analyze circular variables (spatial angles, azimuth, and elevation). To describe circular variables, mean circular direction and 95% confidence interval (CI) values were reported. Nonuniformity of the circular variable distribution was confirmed by the Rayleigh test and the Kuiper test for all studied circular variables. A paired comparison of circular variables was conducted using Hotelling’s paired test. The circular–circular correlation coefficients between two circular variables were calculated by the Fisher and Lee method. The circular–linear correlation coefficients were calculated by the Fisher, Mardia, and Jupp method. The Watson U-square statistic and the Kuiper statistics were used for two-sample tests for circular variables. To account for multiple tests in correlation analyses, the correlation was considered statistically significant if the p-value was less than 0.001.

To determine associations of demographic and clinical characteristics with changes in T and QRS vectors over the course of seven days, we conducted a longitudinal analysis and constructed two sets of mixed models. One set of models was built to predict changes in the direction and magnitude of the T area vector, separately in the AAI (VS) mode and the DDD (VP) mode, respectively. Another set of models was built to predict changes in the direction and magnitude of the QRS area vector in the DDD (VP) mode.

As there was prominent person-to-person variability in QRS and T vector changes, we constructed mixed models with a random slope and intercept. The Hausman specification test confirmed the consistency of random effect estimates for all models. We used an unstructured covariance structure. A likelihood ratio test confirmed a better model fit for a random slope for all models. As recommended for the analysis of circular variables, we transformed them by doubling their value, then adding 360°.^19^ For reporting, we transformed them back.

To test our hypothesis that preexistent remodeling can affect the development of CM, we constructed two models. Model 1 was adjusted for age, sex, and two major causes of preexistent CM (a history of MI and VT). Model 2 was adjusted for other known factors in addition to those in model 1 affecting cardiac structural and electrical remodeling, such as LVEF, a history of diabetes, hypertension, the use of angiotensin-converting enzyme inhibitors (ACEi) or angiotensin receptor blockers (ARBs), and class III antiarrhythmic (AA) drugs. To adjust for possible unmeasured confounders (eg, due to differences in the location of ECG leads in days 1 and 7, or unmeasured disease-related factors), both models 1 and 2 were adjusted for longitudinal changes in the corresponding QRS variable. Also, the model of T (and SVG) azimuth change was adjusted for the QRS azimuth change, the model of T (and SVG) elevation change was adjusted for the QRS elevation change, and the model of T area change was adjusted for the QRS area change. In addition, we tested the hypothesis that T azimuth (and T area) changes during abnormal activation (in the DDD mode) are associated with QRS azimuth (and QRS area) changes, adjusting for the same confounders in models 1 and 2. In mixed models, a p-value of less than 0.05 was considered to be statistically significant. Statistical power calculation is described in **Appendix 1**.^20^

STATA MP 16 (StataCorp LP, College Station, TX, USA) and Oriana-Circular Statistics 4 (Kovach Computing Services, Pentraeth, Wales, UK) were used for statistical analyses. PASS (NCSS, LLC, Kaysville, UT, USA) was used for statistical power calculation.

## Results

### Study population

The clinical characteristics of the study participants are shown in **Table 1**. Most of the study participants were men with ischemic cardiomyopathy and ICDs implanted for primary prevention of sudden cardiac death. Of note, 85% had a history of nonsustained (NS) VT. The vast majority of participants were on β-blockers and ACEi/ARBs, while one-third of participants were receiving class III AA medications (sotalol or amiodarone). During the seven study days, all study participants experienced constant RV pacing; the average percentage of RV pacing was 99.73% ± 0.23% (range: 99.3%–100%).

### Development of cardiac memory

For CM, the mean angle (*μ*) was 67.5° (95% CI: 47.6°–87.4°) median angle was 58.2°, length of the mean vector r-value was 0.727, concentration (κ) value was 2.2, circular variance was 0.27, and the circular SD value was 45.7°.

During paired comparison, there were no differences in the R–R’ interval across all four recordings **(Table 2)**. At baseline, in the APVS beat, the QRS vector was pointed to the left and slightly backward, whereas the T vector was pointed straight forward (indicating preceding electrical remodeling), resulting in a wide baseline QRS–T angle.

VP on day 1 caused QT prolongation, QRS widening, rotation of the QRS vector further backward and up, rotation of the T vector down and leftward, and further widening of the QRS–T angle. Notably, the direction of Wilson’s SVG vector did not change, whereas its magnitude slightly increased. The magnitudes of the QRS and T vectors and areas also increased **(Table 2)**.

**Figure 2:**
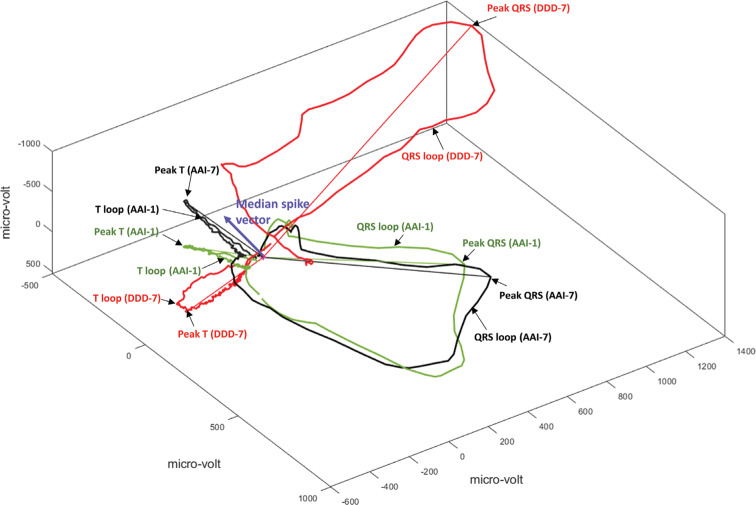
A representative example of QRS and T loops with corresponding peak vectors in median beats recorded at baseline (AAI-1; green) and after the development of CM (AAI-7; black, and DDD-7; red). The median pacing spike vector is shown as a purple arrow. CM: cardiac memory.

After seven days of VP and return to normal activation, the QRS vector returned to the same direction as that seen in conjunction with the APVS baseline beat **(Figure 2)**. QT and QRS intervals, QRS–T angle, and SVG magnitude in AAI-7 did not differ from those in AAI-1. As expected, CM on the APVS beat was manifested by prominent T-vector magnitude enlargement, increased SVG and SAI QRST, and dramatic changes in the T vector direction (turned sharply to the right and upward) and SVG vector direction (turned upward and backward).

On the seventh day, we observed very similar differences between APVS and APVP beats as already seen on the first study day. Neither the magnitude nor direction of Wilson’s SVG differed between APVS and APVP beats.

The comparison of DDD-1 and DDD-7 beats revealed no differences in the direction or magnitude of the QRS vector, the direction of the T vector, or the QRS–T angle. However, the direction of the SVG vector was changed dramatically (turned upward and backward) and the magnitudes of T, SVG, and SAI QRST were significantly decreased.

### Baseline repolarization characteristics associated with cardiac memory

**Figure 3:**
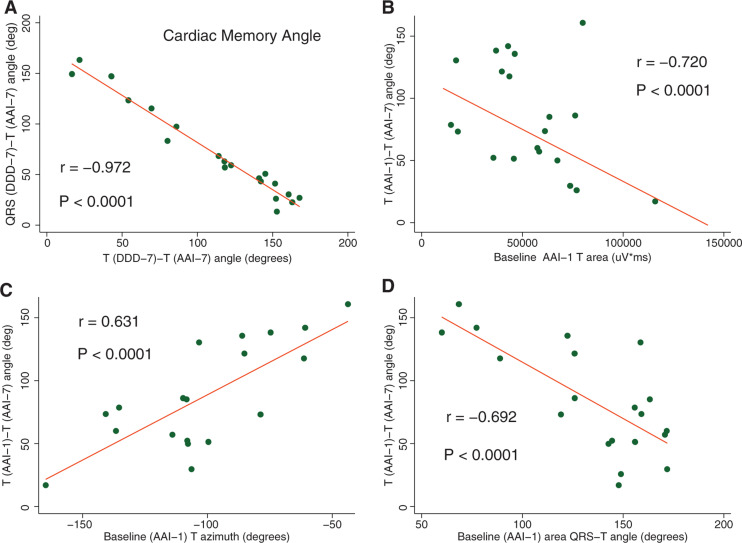
Repolarization memory. **A:** Scatterplot of QRS_DDD-7_–T_AAI-7_ angle (y-axis) against the T_DDD-7_–T_AAI-7_ angle (x-axis). **B–D**: Scatterplots of T_DDD-7_–T_AAI-7_ angle (y-axis) against the T_AAI-1_ area (x-axis), T_AAI-1_ azimuth (x-axis), and QRS–T_AAI-1_ angle, respectively. A linear best-fit line is shown.

The CM angle perfectly correlated with the T_AAI-7_–T_DDD-7_ angle that reflects the difference in repolarization in two different activation patterns after the development of CM **(Figure 3)**. Correlations between the CM (QRS_DDD-7_–T_AAI-7_) angle and T_AAI-1_–T_AAI-7_ (r = −0.198), T_DDD-1_–T_DDD-7_ (r = −0.081), and T_AAI-1_–T_DDD-1_ (r = −0.373) angles, respectively, were weak and nonsignificant.

The T_AAI-1_–T_AAI-7_ angle negatively correlated with the baseline T_AAI-1_ peak magnitude and T_AAI-1_ area and positively correlated with both the T_AAI-1_ peak azimuth and T_AAI-1_ area azimuth **(Figure 3)**. Meanwhile, the baseline QRS–T_AAI-1_ angle was negatively correlated with the T_AAI-1_–T_AAI-7_ angle **(Figure 3)**.

### Baseline characteristics associated with ventricular activation pattern during right ventricular pacing

The difference in ventricular activation between VS and VP QRS vectors as measured by the QRS_AAI-7_–QRS_DDD-7_ angle was, on average, 77.4°, while the length of the mean vector was 0.927 and the median angle was 82.6° (95% CI: 67.7°–87.2°). In the paired analysis, there was no difference between the QRS_AAI-7_–QRS_DDD-7_ and QRS_AAI-1_–QRS_DDD-1_ angles. The degree of possible error due to variations in ECG electrode placement was less than 10°. Meanwhile, the mean QRS_AAI-1_–QRS_AAI-7_ angle was 7.5°, the length of the mean vector was 0.997, and the median angle was 6.5° (95% CI: 5.5°–9.5°). Finally, the mean QRS_DDD-1_–QRS_DDD-7_ angle was 9.6°, the length of the mean vector was 0.976, and the median angle was 5.0° (95% CI: 4.1°–15.1°).

**Figure 4:**
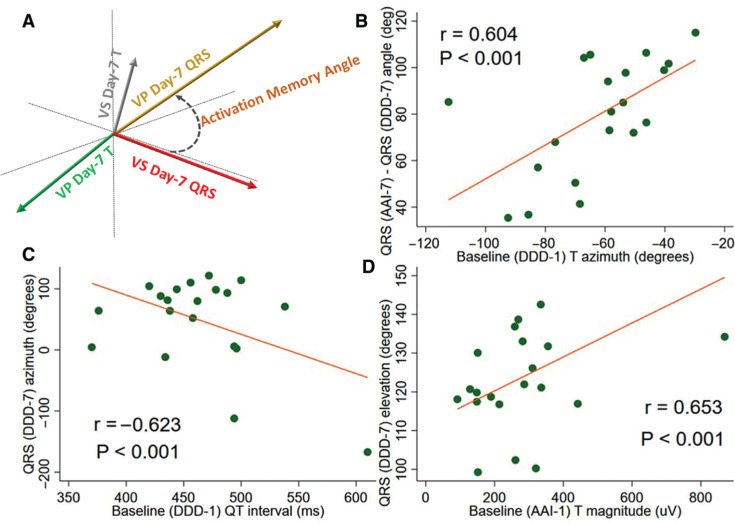
Activation memory. **A:** Schematic of activation memory angle. **B:** Scatterplot of the QRS_AAI-7_–QRS_DDD-7_ angle (y-axis) against the T_DDD-1_ azimuth. **C:** Scatterplot of the QRS_DDD-7_ azimuth (y-axis) against the QT_DDD-1_ interval. **D:** Scatterplot of QRS_DDD-7_ elevation against T_AAI-1_ magnitude. A linear best-fit line is shown.

We observed a significant correlation between baseline APVP repolarization characteristics and differences in ventricular activation in the AAI and DDD modes after CM had developed as measured by the QRS_AAI-7_–QRS_DDD-7_ angle. The baseline APVP T_DDD-1_ azimuth correlated with the QRS_AAI-7_–QRS_DDD-7_ angle **(Figure 4)** but not the QRS_AAI-1_-QRS_DDD-1_ angle (r = 0.355; p > 0.05). The baseline QT_DDD-1_ interval was moderately strongly correlated with the QRS_DDD-7_ azimuth **(Figure 4)**, whereas the correlation between the QT_AAI-1_ interval and QRS_DDD-7_ azimuth was weak (r = 0.436; p < 0.05). The baseline T_AAI-1_ vector magnitude and T_AAI-1_ area were positively correlated with QRS_DDD-7_ vector elevation on the seventh day during VP **(Figure 4)** but not in the context of normal ventricular conduction (QRS_AAI-7_ vector elevation: r = 0.261 for T_AAI-1_ vector magnitude and r = 0.333 for T_AAI-1_ area).

### Clinical characteristics associated with cardiac memory

**Figure 5:**
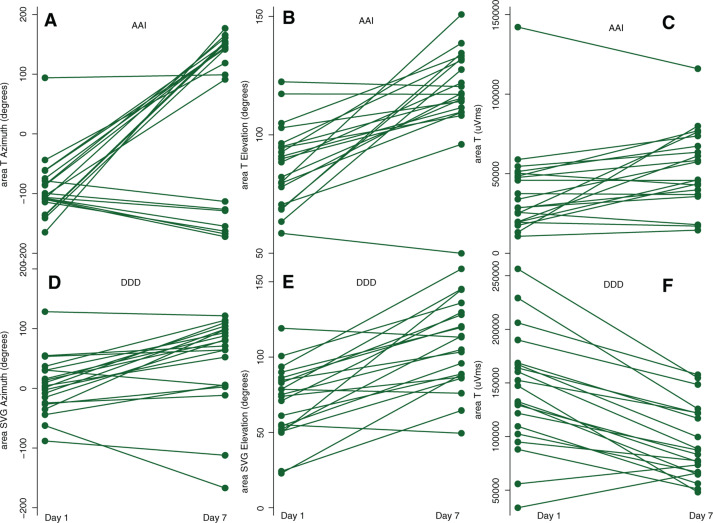
A manifestation of CM in AAI and DDD modes. Time-series line plots show the change in repolarization from day 1 to day 7, for every study participant. Top panels show the changes in T azimuth, T elevation, and area T in the AAI mode. The bottom panels show the changes in SVG azimuth, SVG elevation, and area T in the DDD mode.

We observed prominent person-to-person variability in the changes in T and SVG vectors that manifested CM **(Figure 5)**. The statistical power of the mixed models was sufficient, with few exceptions (see **Table S1** in **Appendix 1**). Mixed model analysis results showed that, in the AAI mode, T azimuth displayed the most dramatic changes from day 1 to day 7 **(Table 3)**. A history of MI and female sex were associated with a significant opposite effect on T azimuth, counteracting the development of CM. Changes in T elevation were associated only with a change in QRS elevation, not with any clinical or demographic characteristics. A history of MI, a history of VT, and a history of class III AA use were associated with T magnitude changes **(Table 3)**. In the DDD mode, a history of VT was associated with changes in SVG azimuth during the seven days of follow-up. A history of MI, VT, and female sex were associated with changes in SVG elevation, reducing the degree of CM manifestation **(Table 3)**. Similarly, female sex and VT history were associated with T magnitude changes in the DDD mode, offsetting the development of CM.

In an adjusted mixed-model analysis, we detected changes in ventricular activation as a manifestation of CM. In DDD mode, we observed significant changes in QRS azimuth and area occurring from day 1 to day 7 **(Table 4)**. Female sex and a history of MI were associated with a reduction in QRS azimuth changes, whereas diabetes and LVEF worsening were associated with greater QRS azimuth changes; similar associations were observed for the QRS area. In AAI mode, no statistically significant changes in the QRS vector direction and magnitude from day 1 to day 7 were observed.

## Discussion

In this prospective study of CM in ICD patients with preexistent cardiac remodeling, we observed several novel findings. First, we showed that preexistent cardiac remodeling due to MI and VT significantly affects the degree of CM in response to RV pacing. Second, we, for the first time, demonstrated the existence of sex differences in CM development. After adjustment for the type of cardiomyopathy, degree of LV dysfunction, use of medications, and major cardiovascular risk factors (hypertension and diabetes), women with preexistent electrical remodeling developed less new CM as compared with men. Third, we noticed that, in participants with preexistent cardiac remodeling, the CM is associated with significant changes in cardiac activation. While repolarization memory “remembers” abnormal activation after baseline activation has been restored, activation memory “remembers” baseline activation during altered (VP) activation, striving for a smaller angle between the QRS vectors of baseline and altered (VP) activation. Further studies of repolarization and activation memory are needed to understand the mechanisms and clinical significance of CM.

### Measurement of cardiac memory on vectorcardiogram

Rosenbaum, with colleagues, was the first to introduce the term “cardiac memory” in the year 1982.^21^ Their work demonstrated how an altered ventricular activation path (or vector) could change the vector of cardiac repolarization. VCG has apparent advantages over ECG in the characterization of cardiac vectors, and, therefore, VCG was adopted very early for the characterization of CM. Plotnikov et al.^22^ quantified CM as “a function of amplitude and angle changes of the T-wave vector and expressed as displacement (in mV) between frontal plane T vector peaks during atrial pacing at baseline and after memory induction.” Such a definition involves the projection of three-dimensional vectors on a single frontal plane, producing a two-dimensional measure. Furthermore, limiting CM’s definition by a single measure does not allow for full characterization of the complex phenomenon of CM.

In this work, we expanded CM quantification and included VCG metrics that have sound biophysical meaning, such as Wilson’s SVG and SAI QRST, together composing global electrical heterogeneity (GEH).^23,24^ SVG is a VCG metric that measures the magnitude and direction of a vector, which points towards the area with the shortest duration of the excited state.^25^ Moreover, SVG reflects the magnitude and direction of the steepest gradient between the areas of the heart with the longest and the shortest recovery time.^23^ SAI QRST and iVMQT are scalar measures of SVG,^16,26^ reflecting electrical dyssynchrony.^27,28^ Acute administration of AA drugs (eg, dofetilide, quinidine, verapamil, and ranolazine) rapidly changes GEH in correlation with the plasma drug levels.^29^ Furthermore, SVG azimuth correlates with a history of drug-induced torsades de pointes independent of the corrected QT interval.^30^ Altogether, these GEH facts support the usefulness of GEH for the characterization of CM.

Importantly, we implemented the fundamental meaning of CM as a phenomenon defined by the repolarization vector following an altered activation vector by measuring a three-dimensional angle between them. We used both area-based and peak-based three-dimensional QRS, T, and SVG vectors and angles. Such a comprehensive characterization of the CM phenotype allowed us to make several novel observations. Notably, we used the physiologically sound definition of the VCG origin point,^15^ which improves the accuracy and physiological interpretation of vectors. We have provided open-source software code for all VCG measurements that can improve the reproducibility and comparability of future studies.

### Spatial ventricular gradient reflects cardiac memory

It was previously postulated that CM is neither a primary nor secondary T-wave change.^6,21^ Our results one more time^31^ confirmed Wilson’s ventricular gradient concept, suggesting that the SVG is largely independent of the ventricular activation sequence (ie, no difference between SVG_AAI-7_ and SVG_DDD-7_). SVG is determined by the heterogeneity in the whole area under the action potential across the heart rather than by heterogeneity in the action-potential duration alone.^23^ The SVG vector tracked the development of CM better than the T vector **(Table 2)**, especially in the DDD mode. Thus, a change in the SVG direction can be used to assess CM in the context of persistently altered activation (eg, during continuous VP, bundle branch block).

### Preexistent cardiac remodeling is associated with the degree of repolarization memory

We observed that the more abnormal the baseline repolarization was, the less repolarization memory developed in response to VP. Also, the wider the baseline spatial QRS–T angle was, the less repolarization memory developed, while, the larger the baseline T area was, the less repolarization memory developed. Similarly, the more abnormal the direction of the baseline T vector, the less repolarization memory developed. An abnormal (rightward and forward) direction of the T vector, large magnitude of T, and wide QRS–T angle are well-known signs of earlier CM or cardiac electrical remodeling. A more negative T vector azimuth in CM patients with DDD RV pacing^32^ is consistent with our finding of less repolarization memory with more negative T vector azimuth **(Figure 2)**. Accordingly, in the mixed model analyses adjusted for a change in cardiac activation between days 1 and 7, LV systolic function, known risk factors, and use of medications known to affect the development of cardiac memory, a history of MI and VT strongly counteracted repolarization changes. A history of MI and VT nearly completely canceled the manifestation of CM in both the AAI and DDD modes. Thus, the robust development of typical CM in response to RV pacing suggests an absence or a minimal degree of preexistent cardiac remodeling. In contrast, the weak repolarization response to RV pacing implies that repolarization ion channels in ventricular cardiomyocytes had already been remodeled, as shown in many in vitro studies,^33,34^ and display a saturation of response. Our finding is consistent with prior CM studies in HF patients undergoing biventricular pacing.^11,12^

### Sex differences in cardiac memory

Previous studies using animal models^34–36^ or human subjects^8,10^ did not investigate sex differences in CM. Interestingly, in our study, sex was strongly associated with the degree of CM manifestation. In fully adjusted mixed models, female sex was strongly and independently associated with a significantly smaller amount of CM. Female sex nearly completely canceled manifestation of CM in both AAI and DDD modes **(Table 3)**. The finding of sex differences in CM is novel and has an important clinical significance. Women demonstrate a reduced expression of potassium channels, resulting in decreased rapid and slow delayed rectifier K^+^ currents, inward rectifier current, and transient outward current.^37,38^ Estrogens inhibit the rapid delayed rectifier current, increase the L-type calcium current, the sodium–calcium exchange current, and calcium release mediated by the ryanodine receptor.^39^

Moreover, it was shown that women have less repolarization reserve as compared with men.^40^ In our study population, RV pacing is a second hit, following after the first hits, which were defined by the study inclusion criteria (ie, cardiomyopathy, history of VT). Postmenopausal women are characterized by diminished repolarization reserve.^41^ After menopause, estradiol levels decline, but a small estradiol concentration persists due to estrogen production by adipose tissue. At the same time, after menopause, there is no progesterone. Without the protective effects of progesterone, the *I*_Kr_-blocking effect of estradiol further reduces the repolarization reserve, resulting in an increase in arrhythmic events after the onset of menopause.^42^ Reduced repolarization reserve can explain why women reach a level of saturation of CM response sooner than men, as observed in this study.

### “Activation memory” is another manifestation of cardiac memory

A large number of previous studies have described CM as a repolarization phenomenon.^8,10,33,36,43^ In this study, we, for the first time, described the manifestation of “activation memory.” Repolarization memory manifests by the T_AAI-7_ vector aligning with an altered activation (QRS) vector; that is, repolarization “remembers” abnormal activation. In turn, activation memory manifests during continued altered activation by an altered activation vector striving to align with a baseline activation vector. Activation memory is likely a compensatory mechanism attempting to minimize dyssynchrony developing in response to sustained altered activation. Activation and repolarization are two sequential phases of a repeated cycle, one following after the other. The development of repolarization memory affects refractoriness in ventricles, which, in turn, affects the way that ventricles are activated. Further investigations of activation memory mechanisms and their clinical significance are needed.

### Limitations

The study size was small and the study population was heterogeneous. Validation of the study findings in a larger study is required. Nevertheless, the statistical power of the analyses was sufficient. Moreover, this study is the largest study of CM in patients with ICDs. We did not assess the changes in the mechanical function and myocardial performance from day 1 to day 7 and their correlations with CM in patients on and off ACEi/ARBs, which should be assessed in future studies.

## Conclusion

CM exhibits saturation behaviors. Preexistent electrical remodeling (eg, in response to previous MI, or VT history as a first hit) is associated with a weak repolarization memory response to a second hit (RV pacing). Women reach CM saturation more easily than men do, likely due to their reduced repolarization reserve.

## Figures and Tables

**Table 1:** Clinical Characteristics of the Study Participants

Characteristic	All Participants (n = 20)
Age (SD), y	72.6 (11.1)
Female sex, n (%)	4 (20)
%VP over seven follow-up days (SD)	99.7 (0.2)
LVEF (SD), %	31.7 (7.6)
Primary prevention ICD, n (%)	16 (80)
NYHA class II, n (%)	10 (50)
History of coronary heart disease, n (%)	17 (85)
History of myocardial infarction, n (%)	15 (75)
Location of infarction (% of patients with MI Hx), n (%)	
Anterior, n	4 (27)
Inferoposterior, n	9 (60)
Multiple (inferior and anterior, anterior and posterolateral)	2 (13)
History of revascularization, n (%)	13 (65)
Hypertension, n (%)	10 (50)
Diabetes, n (%)	7 (35)
Atrial fibrillation, n (%)	9 (45)
History of nonsustained ventricular tachycardia, n (%)	17 (85)
History of sustained ventricular tachycardia, n (%)	4 (20)
Class III AA medications, n (%)	6 (30)
β-blocker therapy, n (%)	19 (95)
Digoxin therapy, n (%)	4 (20)
Calcium channel blocker therapy, n (%)	2 (10)
Diuretic therapy, n (%)	9 (45%)
ACE inhibitor/angiotensin receptor blocker therapy, n (%)	16 (80)

AA: antiarrhythmic; ACE: angiotensin-converting enzyme; ICD: implantable cardioverter-defibrillator; LVEF: left ventricular ejection fraction; MI Hx: myocardial infarction history; NYHA: New York Heart Association; SD: standard deviation; VP: ventricular pacing.

**Table 2:** CM Development

ECG and VCG Parameters	AAI-1	DDD-1	AAI-7	DDD-7	P_AAI1–AAI7_	P_DDD1–DDD7_	P_AAI1–DDD1_	P_AAI7–DDD7_
R–R interval (SD), ms	830 (125)	825 (119)	833 (122)	825 (118)	0.406	0.926	0.456	0.203
QT interval (SD), ms	407 (45)	465 (53)	411 (53)	456 (43)	0.480	0.204	< 0.0001*	< 0.0001*
QRS duration (SD), ms	111 (17)	162 (23)	113 (15)	167 (20)	0.492	0.263	< 0.0001*	< 0.0001*
Spatial QRS area (SD), mV*ms	50 (27)	120 (41)	50 (24)	120 (36)	0.611	0.902	< 0.0001*	< 0.0001*
Spatial peak QRS vector magnitude (SD), mV	1.31 (0.41)	1.45 (0.43)	1.31 (0.35)	1.42 (0.40)	0.907	0.909	0.046*	0.187
Spatial area QRS vector azimuth (95% CI), °	37 (25–49)	99 (90–108)	37 (24–49)	104 (96–111)	0.198	0.384	< 0.0001*	< 0.0001*
Spatial peak QRS vector azimuth (95% CI), °	32 (18–46)	98 (89–106)	31 (17–44)	102 (95–110)	0.964	0.326	< 0.0001*	< 0.0001*
Spatial area QRS vector elevation (95% CI), °	82 (74–90)	122 (117–128)	78 (70–86)	124 (117–130)	0.058	0.202	< 0.0001*	< 0.0001*
Spatial peak QRS vector elevation (95% CI), °	81 (73–88)	121 (116–126)	78 (71–86)	124 (118–130)	0.126	0.160	< 0.0001*	< 0.0001*
Spatial T area (SD), mV*ms	39 (29)	142 (55)	54 (25)	94 (35)	0.007*	< 0.0001*	< 0.0001*	0.0002*
Spatial peak T vector magnitude (SD), mV	0.28 (0.17)	0.91 (0.29)	0.38 (0.14)	0.64 (0.17)	0.003*	< 0.0001*	< 0.0001*	0.0001*
Spatial area T vector azimuth (95% CI), °	−102 (−117 to −88)	−65 (−73 to −57)	166 (156 to −175)	−71 (−79 to −62)	< 0.0001*	0.123	< 0.0001*	< 0.0001*
Spatial peak T vector azimuth (95% CI), °	−92 (−109 to −74)	−63 (−71 to −54)	161 (140–182)	−67 (−77 to −58)	< 0.0001*	0.168	< 0.0001*	< 0.0001*
Spatial area T vector elevation (95% CI), °	87 (80–95)	56 (50–62)	118 (110–126)	55 (48–61)	< 0.0001*	0.423	< 0.0001*	< 0.0001*
Spatial peak T vector elevation (95% CI), °	82 (73–92)	58 (52–63)	119 (111–128)	55 (50–61)	< 0.0001*	0.376	< 0.0001*	< 0.0001*
Wilson’s (area) SVG (SD), mV*ms	36 (22)	45 (31)	44 (17)	42 (14)	0.212	0.615	0.027*	0.209
Spatial peak SVG vector magnitude (SD), mV	0.28 (0.17)	0.91 (0.29)	0.38 (0.14)	0.64 (0.17)	0.003*	< 0.0001*	< 0.0001*	0.0001*
Spatial area SVG vector azimuth (95% CI), °	7 (−15 to 29)	1 (−18 to 20)	91 (78–105)	76 (52–101)	< 0.0001*	< 0.0001*	0.308	0.118
Spatial peak SVG vector azimuth (95% CI), °	27 (12–43)	67 (52–81)	44 (30–57)	91 (82–101)	0.001*	0.003*	< 0.0001*	< 0.0001*
Spatial area SVG vector elevation (95% CI), °	69 (60–78)	70 (60–81)	107 (96–118)	108 (95–121)	< 0.0001*	< 0.0001*	0.510	0.910
Spatial peak SVG vector elevation (95% CI), °	78 (70–85)	112 (106–119)	84 (78–91)	122 (114–130)	0.001*	0.016*	< 0.0001*	< 0.0001*
SAI QRST (SD), mV*ms	157 (74)	415 (141)	181 (61)	329 (90)	0.006*	0.0001*	< 0.0001*	< 0.0001*
Vector magnitude QT integral (SD), mV*ms	106 (50)	268 (91)	118 (42)	219 (66)	0.022*	0.0001*	< 0.0001*	0.0001*
Spatial area QRS–T angle (95% CI), °	136 (121–151)	164 (159–169)	131 (120–141)	168 (165–171)	0.107	0.218	0.0005*	< 0.0001*
Spatial peak QRS–T angle (95% CI), °	115 (94–135)	161 (156–166)	123 (114–134)	166 (162–169)	0.435	0.025*	0.0001*	< 0.0001*

CI: confidence interval; SAI QRST: sum of the absolute QRST integral; SD: standard deviation; SVG: spatial ventricular gradient; VCG: vectorcardiogram.

*Statistically significant

**Table 3:** Demographic and Clinical Characteristics Associated with CM

	Characteristic	Outcome Variables in AAI Mode						Outcome Variables in DDD Mode					
		Area T Azimuth (95% CI), °	p-value	Area T Elevation (95% CI), °	p-value	T Area (95% CI), mV*ms	p-value	Area SVG Azimuth (95% CI), °	p-value	Area SVG Elevation (95% CI), °	p-value	T Area (95% CI), mV*ms	p-value
M1	CM measured by outcome (change days 1–7)	+132 (81–182)*	< 0.0001*	+29 (20–37)*	< 0.0001*	+15 (7–24)*	0.001*	+45 (21–69)*	< 0.0001*	37 (26–50)*	< 0.0001*	−48 (−60 to −35)*	< 0.0001*
M2		+132 (80–184)*	< 0.0001*	+29 (21–38)*	< 0.0001*	+15 (6–24)*	< 0.0001*	+45 (23–69)*	< 0.0001*	37 (26–48)*	< 0.0001*	−48 (− 60 to −36)*	< 0.0001*
M1	Corresponding QRS variable**	−0.4 (−1.3 to 0.4)	0.297	−0.5 (−0.9 to −0.1)*	0.008	+0.5 (0.1–0.8)*	0.005*	−0.04 (−0.8 to 0.7)	0.916	+0.6 (−0.1 to 1.2)	0.115	+0.9 (0.6–1.1)*	0.0001*
M2		−0.4 (−1.4 to 0.7)	0.501	−0.3 (−0.7 to 0.06)	0.104	+0.5 (0.3–0.8)*	< 0.0001*	−0.2 (−1.1 to 0.6)	0.575	+0.5 (−0.2 to 1.3)	0.150	+0.7 (0.5–1.0)*	< 0.0001*
M1	Age, per 1 year	+3 (−0.8 to 6)	0.132	+0.01 (−0.5 to 0.5)	0.967	+0.2 (−0.4–0.8)	0.570	+0.3 (−2 to 2)	0.812	+0.3 (−0.5 to 1.2)	0.449	−0.1 (−1 to 1)	0.865
M2		+4 (0.4–8.5)*	0.033*	−0.2 (−0.8 to 0.4)	0.503	0.2 (−0.5–0.9)	0.499	+0.4 (−2.3 to 3.2)	0.754	0.3 (−0.8 to 1.4)	0.617	+0.3 (−0.7 to 1.4)	0.508
M1	Female vs. male sex	−110 (−201 to −18)*	0.019*	−10 (−26 to 6)	0.231	+7 (−19 to 32)	0.615	−52 (−115 to 12)	0.110	−45 (−72 to −18)*	0.001	+44 (15–72)*	0.002*
M2		−162 (−268 to −55)*	0.003*	−2 (−19 to 16)	0.846	+4 (−18–26)	0.703	−51 (−128 to 26)	0.192	−43 (−76 to −11)*	0.009	+32 (3–61)*	0.029*
M1	History of MI	−124 (−227 to −21)*	0.018*	+5 (−15 to 24)	0.638	−15 (−32 to 3)	0.095	−17 (−75 to 41)	0.559	−24 (−46 to −2)*	0.034	+5 (−21 to 31)	0.696
M2		−180 (−320 to −40)*	0.011*	+19 (−1 to 40)	0.065	−27 (−47 to −8)*	0.006*	−31 (−108 to 47)	0.437	−20 (−49 to 10)	0.188	+0.5 (−29 to 30)	0.969
M1	History of NSVT	−61 (−156 to 34)	0.207	−6 (−22 to 9)	0.432	+22 (0.8–44)*	0.042*	−57 (−122 to 7)	0.083	−30 (−55 to −4)*	0.022	+40 (11–69)*	0.006*
M2		−70 (−165 to 26)	0.153	−6 (−21 to 9)	0.426	+27 (4–46)*	0.007*	−74 (−143 to −5)*	0.034*	−27 (−56 to 0.8)	0.057	+38 (13–63)*	0.003*
M2	Class 3 AA drugs	+1.2 (−74 to 76)	0.975	+8 (−2 to 18)	0.137	−15 (−26 to −4)*	0.010*	+19 (−105 to 253)	0.420	−1 (−19 to 18)	0.950	+13 (−8 to 33)	0.228
M2	ACEi/ARB drugs	+25.9 (−50.2 to 102)	0.504	−10.3 (−21.9 to1.2)	0.080	+0.7 (−13 to 14)	0.918	+18 (−36 to 72)	0.510	−12.2 (−32.5 to 8.2)	0.241	−11.7 (−32 to 8)	0.253

AA: antiarrhythmic; ACEi: angiotensin-converting enzyme inhibitor; ARB; angiotensin receptor blocker; LVEF: left ventricular ejection fraction; MI: myocardial infraction; NSVT: nonsustained ventricular tachycardia; SVG: spatial ventricular gradient; VT: ventricular tachycardia.

Model 1 (M1) was adjusted for the corresponding T variable, age, sex, history of MI, and history of NSVT. Model 2 (M2) was adjusted for the same plus for LVEF, history of diabetes, hypertension, VT, ACEi/ARBs, and class III AA drugs.

*Statistically significant

*Corresponding QRS variable for each model: area QRS azimuth for area T azimuth and area SVG azimuth, area QRS elevation for area T elevation and area SVG elevation, QRS area for T area models.

**Table 4:** Demographic and Clinical Characteristics Associated with Changes in Activation (QRS Vector) in DDD Mode

	Characteristic	Outcome Variables in DDD Mode			
		Area QRS Azimuth (95%CI), °	p-value	QRS Area (95% CI), mV*ms	p-value
M1	Activation change (days 1–7)	+10 (4–15)*	< 0.0001*	+28 (17–38)*	< 0.0001*
M2		+10 (5–15)*	< 0.0001*	+24 (13–36)*	< 0.0001*
M1	Corresponding T variable**	+0.9 (0.7–1.1)*	< 0.0001*	+0.6 (0.4–0.8)*	< 0.0001*
M2		+0.9 (0.7–1.0)*	< 0.0001*	+0.5 (0.3–0.7)*	< 0.0001*
M1	Age, per 1 year	+0.1 (−0.3 to 0.6)	0.639	+0.3 (−0.6 to 1.3)	0.523
M2		0.3 (−0.02 to 0.7)	0.066	+0.8 (−0.3 to 1.8)	0.142
M1	Female vs. male sex	−8 (−19 to 5)	0.213	−30 (−60 to 0.1)*	0.051*
M2		−12 (−22 to −2)*	0.023*	−40 (−69 to −10)*	0.009*
M1	History of MI	−9 (−20 to 3)	0.137	−20 (−45 to 4)	0.101
M2		−17 (−28 to −7)*	0.001*	−28 (−56 to −28)*	0.050*
M1	History of NSVT	+2 (−11 to 15)	0.746	−13 (−43 to 17)	0.405
M2		−3 (−12 to 7)	0.590	−20 (−47 to 6)	0.136
M2	Class 3 AA drugs	+2 (−4 to 8)	0.593	+24 (3–45)*	0.022*
M2	LVEF, per 1%	−0.8 (−1.3 to −0.3)*	0.001*	−0.5 (−1.9 to 0.9)	0.495
M2	Diabetes mellitus	+12 (7–19)*	< 0.0001*	+6.4 (−9.4 to 22.3)	0.427

AA: antiarrhythmic; CI: confidence interval; LVEF: left ventricular ejection fraction; MI: myocardial infraction.

Model 1 (M1) was adjusted for the corresponding T variable, age, sex, history of MI, and history of NSVT. Model 2 (M2) was adjusted for the same plus for LVEF, history of diabetes, hypertension, VT, use of ACEi/ARBs, and class III AA drugs.

*Statistically significant

**Corresponding T variable for each model: area T azimuth for area QRS azimuth, T area for QRS area models.

## Appendix 1

### Supplemental methods

We calculated the statistical power of mixed-model tests for the slope difference in a two-level hierarchical design with random slopes, as formulated by Ahn et al.^20^ as implemented by the PASS 2020 Power Analysis and Sample Size software program.

The longitudinal mixed model equation is as follows:

$${ϒ}_{ij}=\beta_{0}+\xi {\text{X}}_{ij}+\tau {\text{T}}_{ij}+\delta {\text{X}}_{ij}{\text{T}}_{ij}+v_{i}{\text{T}}_{ij}+u_{i}+e_{ij}$$

where

- *Y*_ij_ is the continuous response of the *j*th measurement in the *i*th subject
- β_0_ is the fixed intercept
- X_i_ is an indicator variable for a categorical variable (eg, male or female)
- T_ij_ is the time value
- ξ is the intervention effect at baseline and is expected to be zero
- δ is the difference between the two slopes
- *υ*_i_** is subject-specific slope for the *i*th subject, which is distributed as *N*(0, $\sigma_{T}^{2}$)
- *u*_i_ is a random effect (subject-specific intercept) for the *i*th cluster, distributed as (0, $\sigma_{u}^{2}$)
- *e*_i_ is a random effect for the *j*th subject in the *i*th cluster that is distributed as (0, $\sigma_{e}^{2}$)
- $\sigma_{u}^{2}$ is the variance of the subject random effects
- $\sigma_{e}^{2}$ is variance of the measurement random effects
- $\sigma_{T}^{2}$ is variance of the subject-specific slopes
- σ^2^ is the variance of *Y*, where $\sigma^{2}=\sigma_{e}^{2}/(1-\rho )$
- ρ is the correlation between measurements on the same subject
- *K*_1_ is the number of subjects in group 1
- *K*_2_ is the number of subjects in group 2
- λ is *K*_1_/*K*_2_
- *M* is the number of measurements per subject
- *r*_T_ is ratio of the random slope variance to the sum of the other variance terms, σ^2^

The test of significance of the X_ij_T_ij_ term in the mixed-model analysis is the test statistic of interest. It tests the difference of the two group slopes.

The power was calculated using the following equation:

$$Power=\Phi \left\{\frac{\delta }{\sigma }\sqrt{K_{2}MV(T)/[(1+\lambda^{-1})\{(1-\rho )+r_{T}MV(T)\}]}-\Phi \left(1-\frac{\alpha }{2}\right)\right\}$$

**Supplemental Table 1:** Statistical Power of Mixed-model Analyses for Binary Covariates

Power for	Outcome Variables in AAI Mode			Outcome Variables in DDD Mode				
Characteristic	Area T Azimuth	Area T Elevation	T area	Area SVG Azimuth	Area SVG Elevation	T Area	Area QRS Azimuth	QRS Area
Female vs. male sex	1.00	1.00	0.05	0.40	1.00	1.00	1.00	1.00
History of MI	1.00	1.00	0.21	0.20	0.96	1.00	1.00	1.00
History of NSVT	0.90	1.00	0.16	0.59	0.97	1.00	1.00	0.96
Class 3 AA drugs	0.03	1.00	0.10	0.11	0.04	1.00	1.00	1.00

AA: antiarrhythmic; MI: myocardial infarction; VT: ventricular tachycardia.
